# The Impact of Anhedonia on the Disease Burden of Major Depressive Disorder in the Asia–Pacific Region: A Cross‐Sectional Real‐World Study

**DOI:** 10.1002/npr2.70007

**Published:** 2025-02-26

**Authors:** Keira Herr, Michael Berk, Wei‐Lieh Huang, Tadafumi Kato, Jung Goo Lee, Chong Guan Ng, Zhen Wang, Thomas Webb, Mami Kasahara‐Kiritani, Lawrence Vandervoort

**Affiliations:** ^1^ Janssen Asia Pacific Singapore Singapore; ^2^ School of Medicine Deakin University Melbourne Australia; ^3^ Department of Psychiatry National Taiwan University Hospital Yunlin Branch Yunlin Taiwan; ^4^ Department of Psychiatry and Behavioral Science Juntendo University Graduate School of Medicine Tokyo Japan; ^5^ Department of Psychiatry, College of Medicine, Haeundae Paik Inje University Busan Republic of Korea; ^6^ Paik Institute for Clinical Research Inje University Busan Republic of Korea; ^7^ Department of Psychological Medicine, Faculty of Medicine University of Malaya Kuala Lumpur Malaysia; ^8^ Shanghai Mental Health Center Shanghai Jiao Tong University School of Medicine Shanghai China; ^9^ Janssen Pharmaceutical K.K. Tokyo Japan; ^10^ Oracle Life Sciences Singapore Singapore

**Keywords:** anhedonia, clinical burden, economic burden, major depressive disorder, patient burden

## Abstract

**Aim:**

Anhedonia is a key symptom of major depressive disorder (MDD), however, its burden in patients with MDD is not well understood. We aimed to assess the impact of anhedonia on health‐related quality of life (HRQoL), health‐care resource utilization (HRU), and work productivity in subjects with MDD and anhedonia (MDD‐ANH) compared to subjects with MDD without ANH (MDD non‐ANH).

**Methods:**

A cross‐sectional web‐based survey was conducted across six countries/territories. Adult participants were categorized as MDD‐ANH, MDD non‐ANH, and General Population based on self‐reported MDD diagnosis, Patient Health Questionnaire (PHQ‐9), and Snaith‐Hamilton Pleasure Scale (SHAPS). Multivariate/generalized linear regression modeling (GLMs) and mediation analysis were used to assess anhedonia's impact on HRQoL/function, HRU, and work productivity.

**Results:**

Among 11 383 respondents, 20.1% were identified with MDD (MDD‐ANH: 12.7%; MDD non‐ANH: 7.3%) and 79.9% as General Population. Subjects with MDD‐ANH, compared with MDD non‐ANH demonstrated significantly worse or lower sexual functioning, HRQoL (RAND mental/physical component summary, health state utility (EuroQol) Index scores, all *p* < 0.001), and higher HRU (psychiatrist visits). Work productivity (higher absenteeism/overall work productivity or daily life impairment scores; all *p* < 0.05) was significantly worse in subjects with MDD‐ANH compared with MDD non‐ANH.

**Conclusion:**

Anhedonia in patients with MDD had a significant negative impact on HRQoL, sexual functioning, work productivity, and HRU, emphasizing the need for focus on anhedonia management in MDD patients in the Asia–Pacific region.

## Abstract 2

This cross‐sectional, web‐based survey study was conducted across Australia, China Mainland, Japan, Malaysia, South Korea, and Taiwan, to assess the impact of anhedonia on the health‐related quality of life (HRQoL), health‐care resource utilization (HRU), and work productivity in subjects with major depressive disorder (MDD). Multivariate analysis revealed that MDD subjects with anhedonia had significantly worse sexual functioning HRQoL as well as higher HRU and impairment to work productivity and activity than subjects without anhedonia.

## Graphical abstract

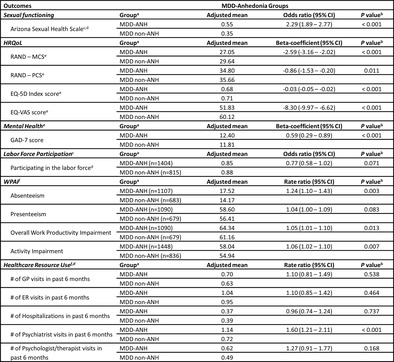

## 1 Introduction

Major depressive disorder (MDD) is a pervasive mental disorder with a substantial disease burden worldwide [1, 2, 3]. A meta‐analysis, based on studies published between 1994 and 2014, found that the point aggregate prevalence of depression in Asia was 16.7%, the second‐highest prevalence globally [4]. MDD is associated with adverse outcomes such as poorer cognitive and social functioning, occupational‐related outcomes, or workplace function, thereby negatively impacting health‐related quality of life (HRQoL) and productivity. The global economic impact of MDD is significant, including the Asia–Pacific region. Patients with MDD incur not just higher health‐care expenses and utilization but also reduced work productivity [5, 6, 7, 8].

Anhedonia, defined as the loss of interest or pleasure in all activities, is experienced by up to 70%–75% of patients with MDD [1, 9, 10, 11, 12]. As per the Diagnostic and Statistical Manual of Mental Disorders Fifth Edition (DSM‐5), anhedonia is a key diagnostic criterion for MDD [1, 12].

Data on the prevalence and burden of anhedonia in the Asia–Pacific is scarce. Although some studies have assessed its impact on social interaction in MDD patients [13, 14], there is limited evidence evaluating the impact on patient‐centric and economic outcomes. Measures of anhedonia like the Snaith‐Hamilton Pleasure Scale (SHAPS) [15] have recently been validated in studies across the Asia–Pacific [16, 17], providing an opportunity to better understand the burden of anhedonia in patients with MDD in this region.

An understanding of the intricate relationship between anhedonia and patient‐centric outcomes would underscore the importance of approaches to manage both overall disease burden and HRQoL in patients with persistent anhedonia. This real‐world study assessed the burden of anhedonia among a nationally representative sample of adults self‐reporting physician‐diagnosed depression in the Asia–Pacific region. Here, we present data on patient‐centric, economic, and clinical burden among adults with MDD with anhedonia (MDD‐ANH) compared to those with MDD without ANH (MDD non‐ANH).

## 2 Methods

### 2.1 Study Design and Data Source

This cross‐sectional, observational, web‐based survey study was conducted in April–May 2023 in patients with MDD‐ANH, MDD non‐ANH, and non‐MDD (referred to as General Population) in Australia, China Mainland, Japan, Malaysia, South Korea, and Taiwan.

The potential respondents were selected from an existing, general‐purpose (i.e., not health‐care‐specific) web‐based consumer panel that is age and gender‐representative of each country/territory. After explicitly agreeing to join the panel, participants receive periodic invitations to online surveys. This study aimed to recruit 350 to 400 MDD patients and 1400 to 1600 General Population participants per country/territory. Respondents who agreed to complete and pass the eligibility screener provided consent and received the 20 to 30 min questionnaire on functioning (including work productivity and activity impairment [WPAI]), HRQoL, and health‐care resource utilization (HRU) were included in the study. The respondents were allowed to quit the survey if they did not wish to continue by closing the web browser and not return; their data were not collected. Respondents received remuneration for their time and participation in the study and were not followed up once they completed the survey.

The study was conducted as per the Declaration of Helsinki and in line with Good Epidemiological Practices as outlined by the International Society for Pharmacoeconomics and Outcomes Research. The study protocol and study material (questionnaire) were submitted to and approved by the Toukekai Kitamachi Clinic Ethics Review Board (ERB) (approval number: EJP09413) for the conduct of the study in Japan. The study protocol (along with study material [questionnaire]) was submitted to the Pearl Institutional Review Board (for conduct of the study in Australia, China Mainland, Malaysia, South Korea, and Taiwan) and was determined to be exempt as per regulations: 45 CRF 46.104(b) (2) Tests, Surveys, Interviews (IRB number: 023‐0025). Only deidentified data was collected and analyzed. Participants in this study had only provided informed consent to sharing analyzed aggregated data, not individual‐level data, even if anonymized.

### 2.2 Study Population

Respondents (age ≥ 18 years) were self‐reported in this study as MDD‐ANH, MDD non‐ANH, and General Population. MDD‐ANH patients were defined as respondents with a self‐reported physician diagnosis of MDD, a self‐reported Patient Health Questionnaire‐9‐item (PHQ‐9) score ≥ 10, and a SHAPS score > 2. MDD non‐ANH patients were defined as respondents with a self‐reported physician diagnosis of MDD, self‐reported PHQ‐9 score of ≥ 10, and SHAPS score of ≤ 2. General Population respondents were defined as respondents without self‐reported physician diagnosis of MDD and no self‐reported MDD in the past 2 weeks based on a PHQ‐9 score ≤ 4. Adults with self‐reported bipolar disorder or schizophrenia were excluded (Figure S1).

### 2.3 Sample Size

A sample size of 350 to 400 per country/territory was aimed (with 80% power) for the MDD group (per region; 75% with MDD‐ANH), to detect clinically meaningful differences (effect size: 0.2 to 0.5) between MDD‐ANH and MDD non‐ANH groups. Similarly, a sample size of 1400 to 1600 per country/territory, with 80% power, was aimed for the General Population to detect clinically meaningful differences (effect size: 0.01 to 0.2) between the MDD group and the General Population.

### 2.4 Study Measures and Outcomes

#### 2.4.1 Sociodemographic and Health‐Related Characteristics

Sociodemographic (age, sex, race/ethnicity, marital status, education, employment status, household income, health insurance), health (body mass index [BMI], alcohol use, cigarette smoking, exercise activity, and diagnosis of comorbidities [by Charlson comorbidity index, CCI]), and clinical characteristics were collected and included in the analysis.

#### 2.4.2 Depression and Anxiety

PHQ‐9 questionnaire was used to measure depression and its severity [18]. The scale measured the frequency of depression symptoms experienced in the past 2 weeks, on a 4‐point scale (0 = not at all to 3 = nearly every day) which was summed up into a total score (range: 0 to 27). A total score of 5, 10, 15, and 20 represented cutoffs for mild, moderate, moderately severe, and severe depression, respectively. A 14‐item SHAPS score [15] assessed 4 domains of pleasure response or anhedonia and was employed to stratify MDD respondents into MDD‐ANH and MDD non‐ANH using SHAPS cutoff score of > 2 [19, 20, 21]. Higher SHAPS score indicated higher anhedonia.

Generalized Anxiety Disorder Assessment (GAD‐7) assessment was employed to assess the severity of anxiety symptoms over the past 2 weeks. Response options included “not at all,” “several days,” “more than half the days,” and “nearly every day.” Scores ranged between 0 and 21 with scores of 5, 10, and 15 as cutoffs for mild, moderate, and severe anxiety, respectively [22].

The participants' sexual functioning was quantified through a 5‐item Arizona Sexual Experience Scale (ASEX). These scores ranged from 5 to 30 with higher scores indicating more sexual dysfunction [23].

Scales such as PHQ‐9 and GAD‐7 have been translated into multiple languages, which demonstrate good psychometric properties [24, 25, 26]. The subjects were administered with the SHAPS that was translated and validated in Japanese, Malay, and Simplified Chinese [27, 28, 29, 30]. The validated version of SHAPS in Traditional Chinese was not available at the initiation of study for administration in Taiwan and was translated and verified by two certified local language specialists.

#### 2.4.3 Health‐Related Quality of Life and Health‐State Utility

HRQoL was assessed through the RAND‐36, an HRQoL instrument [31]. Two summary scores, physical component summary (PCS) and mental component summary (MCS), were calculated. The total scores ranged on a scale of 0 to 100, with higher scores indicating better HRQoL. A 3‐ to 5‐point difference between the groups is an established minimal clinically important difference [32].

The 5‐level EQ‐5D questionnaire (EQ‐5D‐5L) was also used to measure HRQoL, with an overall index score of 0 (health state equivalent to death) and 1 (perfect health) [33]. Further, the EQ‐VAS was employed in respondents to indicate their self‐rated health from “Worst imaginable health state = 0” to “Best imaginable health state = 100” [34].

#### 2.4.4 Labor Force Participation and WPAI

Data on labor force participation was collected from respondents' self‐reported employment status. Respondents who were of working age (18 to 65 years), employed full time/employed part time/self‐employed/ students were included in the labor force whereas those who were not employed, not of working age, retired, homemakers were not considered.

Work productivity was assessed using the WPAI questionnaire, a 6‐item validated instrument that consisted of four parameters: absenteeism, presenteeism, overall work productivity loss assessed (Only in respondents being full time, part time, or self‐employed), and activity impairment (provided by all respondents) [35].

#### 2.4.5 HRU

HRU (self‐reported) was defined by the number of traditional health‐care provider or general physician (GP) visits, the number of psychiatrist visits, psychologist/therapist visits, and emergency room (ER) visits (“how many times have you been to the emergency room for your own medical condition in the past 6 months?”), and the frequency of hospitalization (“how many times have you been hospitalized for your own medical condition in the past 6 months?”) in the past 6 months.

### 2.5 Statistical Analyses

Comparisons between MDD‐ANH and MDD non‐ANH on all demographic and clinical characteristics were performed. Differences in depression‐specific characteristics between MDD‐ANH and MDD non‐ANH were compared using bivariate analysis. Independent‐sample *t*‐tests were used to compare continuous or discrete variables (e.g., age), and chi‐squared tests to compare categorical variables (e.g., sex) among groups.

A stepwise multivariate analysis approach was used to quantify the impact of anhedonia on MDD on the clinical, patient‐centric, and economic burden outcomes. Each outcome of interest was modeled individually (through generalized linear regression/Binary logistic regression model). An initial three‐group multivariate analysis (MDD‐ANH patients and MDD non‐ANH patients compared with the General Population) correcting for covariates that might contribute to MDD (country/territory, race, sex, age, BMI, smoking habits, alcohol consumption, frequency of exercise, education, employment, and CCI) was first conducted; thereafter, a two‐group comparison (MDD‐ANH vs. MDD non‐ANH) to assess the impact of anhedonia on MDD on the outcomes, adjustment of covariates that might contribute to anhedonia (MDD severity [PHQ‐9 score]) or a selection thereof (Table S1) was conducted.

A mediation analysis quantified the proportion of the MDD with an anhedonia impact mediated by anhedonia on the burden of disease, HRU, and productivity (Table S1 and Figure S2). The analysis was adjusted for covariates such as country/territory, age, sex, and CCI.

Independent‐sample *t*‐tests (or ANOVA, in the case of more than two groups) were used to evaluate unadjusted subgroup differences for continuous variables or to compare continuous/discrete variables. Chi‐squared tests were used to determine significant differences across subgroups for or compare categorical variables. Parameter estimates/exponentiated parameter estimates, standard deviation (SD), 95% confidence intervals (CI), and adjusted odds ratios, rate ratios (OR) or beta‐coefficients are reported. *p* values < 0.05, two‐tailed, were considered statistically significant. All statistical analyses were conducted using IBM SPSS Statistics for Windows, Version 29.0 (IBM Corp., Armonk, NY) and/or R 4.2.2 and/or SAS 9.4.

## 3 Results

**FIGURE 1:**
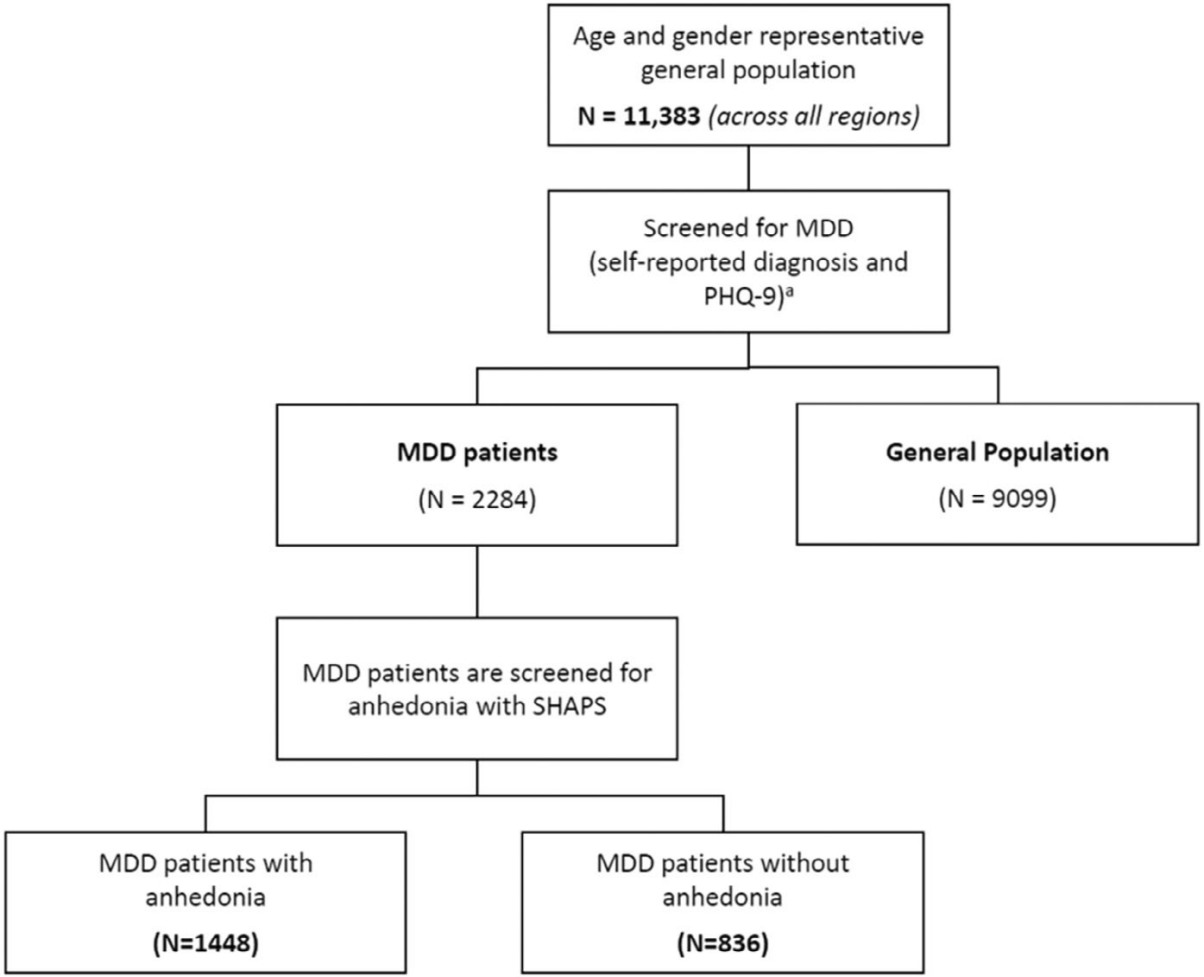
Flow of participants. ^a^Bipolar or schizophrenic patients will be excluded. Abbreviations: ANH, anhedonia; MDD, major depressive disorder; MDD‐ANH, MDD with anhedonia; MDD non‐ANH, MDD without anhedonia; PHQ‐9, Patient Health Questionnaire‐9‐item; SHAPS, Snaith‐Hamilton Pleasure Scale.

A total of 38 623 participants were invited to participate in the study and 27 241 were excluded for incomplete survey or screener, did not provide consent, had self‐reported diagnosis of bipolar disorder or schizophrenia, had self‐reported physician diagnosis of MDD but had PHQ‐9 score < 10, or had no self‐reported physician diagnosis of MDD but had PHQ‐9 score ≥ 10, and overset quotas (Figure S1). Overall, 11 383 eligible participants who had provided informed consent and completed the study across six countries/territories were included in the study, of which 1448 (12.7%) were identified as MDD‐ANH, 836 (7.3%) as MDD non‐ANH, and 9099 (79.9%) were from the General Population (Figure 1).

### 3.1 Demographic and Health Characteristics

A smaller proportion of respondents in the MDD‐ANH compared to MDD non‐ANH were females (52.3% vs. 60.7%; general population: 46.6%), aged 18 to < 35 years (41.8% vs. 47.8%; general population: 23.5%), and attended university/ graduate school (60.0% vs. 65.6; general population 62.7%). The demographics and health characteristics are detailed in Table 1 and Table S2.

**TABLE 1:** Sociodemographic and health characteristics of the MDD‐ANH, MDD non‐ANH, and General Population.

	MDD‐ANH (*n* = 1448)	MDD non‐ANH (*n* = 836)	General population (*n* = 9099)
	*n* (%)	*n* (%)	*n* (%)
S1. Sex			
Male	691 (47.7)	329 (39.4)	4863 (53.5)
Female	757 (52.3)	507 (60.7)	4236 (46.6)
S2. Age category			
18 to < 25	148 (10.2)	113 (13.5)	512 (5.6)
25 to < 35	458 (31.6)	287 (34.3)	1630 (17.9)
35 to < 45	396 (27.4)	253 (30.3)	2211 (24.3)
45 to < 55	264 (18.2)	107 (12.8)	1900 (20.9)
55 to < 65	125 (8.6)	52 (6.2)	1463 (16.1)
≥ 65	57 (3.9)	24 (2.9)	1383 (15.2)
S3. Education			
Primary school	5 (0.4)	5 (0.6)	23 (0.3)
Secondary school	94 (6.5)	61 (7.3)	485 (5.3)
Senior secondary school	272 (18.8)	130 (15.6)	1541 (16.9)
Vocational Education and Training (VET)	191 (13.2)	83 (9.9)	1249 (13.7)
University	739 (51.0)	463 (55.4)	4584 (50.4)
Graduate school or above	130 (9.0)	85 (10.2)	1122 (12.3)
No school	1 (0.1)	1 (0.1)	4 (0.0)
Others	13 (0.9)	6 (0.7)	75 (0.8)
Decline to answer	3 (0.2)	2 (0.2)	16 (0.2)
S4. Employment status			
Employed full time	875 (60.4)	540 (64.6)	5749 (63.2)
Self‐employed	94 (6.5)	63 (7.5)	682 (7.5)
Employed part time	156 (10.8)	96 (11.5)	666 (7.3)
Homemaker	68 (4.7)	32 (3.8)	468 (5.1)
Retired	52 (3.6)	20 (2.4)	909 (10.0)
Student	48 (3.3)	35 (4.2)	279 (3.1)
Long‐term disability^a^	43 (3.0)	16 (1.9)	18 (0.2)
Not employed, but looking for work	70 (4.8)	38 (4.6)	214 (2.4)
Not employed and not looking for work	67 (4.6)	13 (1.6)	193 (2.1)
Short‐term disability^b^	8 (0.6)	3 (0.4)	15 (0.2)
S5. CCI score categories			
0	803 (55.5)	442 (52.9)	5005 (55.0)
1	272 (18.8)	111 (13.3)	1600 (17.6)
2	143 (9.9)	83 (9.9)	1233 (13.6)
3+	230 (15.9)	200 (23.9)	1261 (13.9)
Disease burden scores			
PHQ‐9 score^c^, mean (SD)	16.61 (4.54)	15.08 (4.14)	1.73 (1.42)
SHAPS score^d^, mean (SD)	7.23 (3.28)	0.73 (0.79)	

Abbreviations: ANH, anhedonia; CCI, Charlson comorbidity index; MDD, major depressive disorder; MDD‐ANH, MDD with anhedonia; MDD non‐ANH, MDD without anhedonia; PHQ‐9, Patient Health Questionnaire‐9‐item; SD, standard deviation; SHAPS, Snaith‐Hamilton Pleasure Scale.

^a^ Japan: long‐term leave of absence due to illness of your own (> 3 months).

^b^ Japan: short‐term leave of absence due to illness of your own (< 3 months).

^c^ PHQ‐9 (range 0 to 27); a higher score indicates more severe depression. Scores of 5, 10, 15, and 20 represent cutoffs for mild, moderate, moderately severe, and severe depression, respectively.

^d^ SHAPS (range 0 to 14); a higher score indicates higher levels of anhedonia.

Respondents with MDD‐ANH, versus MDD non‐ANH, had higher PHQ‐9 scores (mean [SD], 16.61 [4.54] vs. 15.08 [4.14]) and SHAPS score (mean [SD], 7.23 [3.28] vs. 0.73 [0.79]) (Table 1).

### 3.2 Sexual Functioning

Respondents with MDD‐ANH reported significantly worse ASEX scores, compared with MDD non‐ANH patients (MDD‐ANH versus MDD non‐ANH: OR [95% CI] 2.29 [1.89 to 2.77]; *p* < 0.001) (Table 2). Furthermore, respondents with MDD‐ANH and MDD non‐ANH scored significantly higher (worse) on ASEX scales versus the General Population (both *p* < 0.001) (Table S3).

**TABLE 2:** Multivariable results for patient‐centric, economic, and clinical outcomes (MDD‐ANH versus MDD non‐ANH).

Outcomes	MDD‐Anhedonia groups			
Sexual functioning	Group^a^	Adjusted mean	Odds ratio (95% CI)	*p* ^b^
Arizona sexual health scale^c^, ^d^	MDD‐ANH	0.55	2.29 (1.89 to 2.77)	< 0.001
	MDD non‐ANH	0.35		

HRQoL	Group^a^	Adjusted mean	Beta‐coefficient (95% CI)	*p* ^b^
RAND‐MCS^e^	MDD‐ANH	27.05	−2.59 (−3.16 to −2.02)	< 0.001
	MDD non‐ANH	29.64		
RAND‐PCS^e^	MDD‐ANH	34.80	−0.86 (−1.53 to −0.20)	0.011
	MDD non‐ANH	35.66		
EQ‐5D Index score^e^	MDD‐ANH	0.68	−0.03 (−0.05 to −0.02)	< 0.001
	MDD non‐ANH	0.71		
EQ‐VAS score^e^	MDD‐ANH	51.83	−8.30 (−9.97 to −6.62)	< 0.001
	MDD non‐ANH	60.12		

Mental health^e^	Group^a^	Adjusted mean	Beta‐coefficient (95% CI)	*p* ^b^
GAD‐7 score	MDD‐ANH	12.40	0.59 (0.29 to 0.89)	< 0.001
	MDD non‐ANH	11.81		

Labor force participation^c^	Group	Adjusted mean	Odds ratio (95% CI)	*p* ^b^
Participating in the labor force^d^	MDD‐ANH (*n* = 1404)	0.85	0.77 (0.58 to 1.02)	0.071
	MDD non‐ANH (*n* = 815)	0.88		

WPAI^f^	Group	Adjusted mean	Rate ratio (95% CI)	*p* ^b^
Absenteeism	MDD‐ANH (*n* = 1107)	17.52	1.24 (1.10 to 1.43)	0.003
	MDD non‐ANH (*n* = 683)	14.17		
Presenteeism	MDD‐ANH (*n* = 1090)	58.60	1.04 (0.99 to 1.09)	0.083
	MDD non‐ANH (*n* = 679)	56.41		
Overall work productivity impairment	MDD‐ANH (*n* = 1090)	64.34	1.05 (1.01 to 1.10)	0.013
	MDD non‐ANH (*n* = 679)	61.16		
Activity impairment	MDD‐ANH (*n* = 1448)	58.04	1.06 (1.02 to 1.10)	0.007
	MDD non‐ANH (*n* = 836)	54.94		

Health‐care resource use^d^, ^f^	Group^a^	Adjusted mean	Rate ratio (95% CI)	*p* ^b^
# of GP visits in past 6 months	MDD‐ANH	0.70	1.10 (0.81 to 1.49)	0.538
	MDD non‐ANH	0.63		
# of ER visits in past 6 months	MDD‐ANH	1.04	1.10 (0.85 to 1.42)	0.464
	MDD non‐ANH	0.95		
# of hospitalizations in past 6 months	MDD‐ANH	0.37	0.96 (0.74 to 1.24)	0.737
	MDD non‐ANH	0.39		
# of psychiatrist visits in past 6 months	MDD‐ANH	1.14	1.60 (1.21 to 2.11)	< 0.001
	MDD non‐ANH	0.72		
# of psychologist/therapist visits in past 6 months	MDD‐ANH	0.62	1.27 (0.91 to 1.77)	0.168
	MDD non‐ANH	0.49		

Abbreviations: ANH, anhedonia; CCI, Charlson comorbidity index; CI, confidence interval; EQ‐5D‐5L, EuroQol 5 Dimension Health Questionnaire; ER, emergency room; GAD‐7, 7‐item Generalized Anxiety Disorder Assessment scale; GP, general physician; MCS, mental component summary; MDD, major depressive disorder; MDD‐ANH, MDD with anhedonia; MDD non‐ANH, MDD without anhedonia; PCS, physical component summary; VAS, visual analogue scale; WPAI, Work productivity and activity impairment.

^a^ MDD‐ANH (*n* = 1448); MDD non‐ANH (*n* = 836).

^b^ *p* value was calculated based on the comparison of MDD‐ANH versus MDD non‐ANH. The analysis was performed Controlling for country, race, sex, age, BMI, frequency of smoking, frequency of consuming alcohol, frequency of exercise, education, employment status, CCI, and PHQ‐9 (Item 1 removed) unless.

^c^ Binary logistic regression model.

^d^ Covariates restricted to age, sex, CCI, and PHQ‐9 (Item 1 removed) due to convergence issues.

^e^ GLM w/ normal distribution and identity link.

^f^ GLM w/ negative binomial distribution and log‐link.

^#^ Number.

Anhedonia and depression were statistically significantly correlated with higher levels of sexual dysfunction, as measured by ASEX. 41.7% of the correlation between depression and sexual dysfunction was mediated by anhedonia (*p* < 0.001) (Table S4).

### 3.3 HRQoL

As compared with MDD non‐ANH respondents, MDD‐ANH respondents scored significantly worse on RAND MCS: OR (95% CI), −2.59 (−3.16 to −2.02), PCS: −0.86 (1.53 to −0.20), EQ‐5D: −0.03 (−0.05 to −0.02), and EQ‐VAS: −8.30 (−9.97 to −6.62); all *p* < 0.001 (Table 2). Respondents with MDD‐ANH and MDD non‐ANH scored significantly worse on RAND MCS scores, RAND PCS scores, health utility scores such as EQ‐5D index scores, and EQ‐VAS scores than the General Population (all *p* < 0.001) (Table S3).

The mediation percentages for the impact of anhedonia on MDD HRQoL outcomes such as RAND MCS and PCS scores, EQ‐5D index, and EQ‐VAS scores were at 15.6% (0.156), 9.9% (0.099), 11.7% (0.117), 27.3% (0.273); all *p* < 0.001 (Table S4).

### 3.4 GAD‐7

The GAD‐7 scores were higher (worse) in MDD‐ANH compared with MDD non‐ANH respondents: OR (95% CI) 0.59 (0.29 to 0.89), *p* < 0.001 (Table 2). Respondents with MDD‐ANH and MDD non‐ANH had significantly worse GAD‐7 scores when compared with the General Population (both *p* < 0.001) (Table S3).

The mediation percentage for the impact of anhedonia on GAD‐7 scores was at 3.6%, *p* < 0.001 (Table S4).

### 3.5 WPAI

MDD‐ANH respondents, versus MDD non‐ANH, experienced significantly more absenteeism (OR 1.24 [95% CI: 1.10 to 1.43]; *p* = 0.003), greater overall work productivity impairment (OR 1.05 [95% CI: 1.01 to 1.10]; *p* = 0.013), and higher impairment in daily activity (OR 1.06 [95% CI: 1.02 to 1.10]; *p* = 0.007) (Table 2).

MDD‐ANH and MDD non‐ANH respondents had significantly lower labor force participation than the General Population (*p* < 0.001 and *p* = 0.055). Patients with MDD‐ANH and MDD non‐ANH, versus General Population, scored significantly higher on absenteeism, presenteeism, overall work productivity impairment, and daily activity impairment (all *p* < 0.001) (Table S3).

The mediation percentages for the impact of anhedonia on labor force participation and WPAI parameters were as follows: labor force participation at 21.0%, absenteeism at 16.2%, presenteeism at 10.4%, overall work productivity impairment at 12.4%, and daily activity impairment at 11.6%; all *p* < 0.001 (Table S5).

### 3.6 HRU

HRU rates (GP/ER/Psychologist visits/hospitalizations) in MDD‐ANH respondents did not differ from MDD non‐ANH (*p* > 0.05) except for the number of psychiatrist visits in 6 months: OR (95% CI), 1.60 (1.21–2.11); *p* < 0.001 (Table 2). HRU was significantly higher in the MDD‐ANH population and MDD non‐ANH versus the General Population, in terms of the number of GP visits, ER visits, psychiatrist visits, psychologist/therapist visits, and frequency of hospitalization in the past 6 months (all *p* < 0.001) (Table S3).

The mediation percentage for the impact of anhedonia on psychiatrists' visits was at 11.9%, *p* = 0.04 (Table S6).

## 4 Discussion

This cross‐sectional study was the first large‐scale study that assessed the patient‐centric, economic, and clinical disease burden among MDD patients with anhedonia (MDD‐ANH) compared to adults with MDD non‐ANH across six countries/territories in the Asia–Pacific region. Notably, 20.1% of respondents were identified as MDD in this study, which is relatively higher than previously reported in Asia–Pacific [36]. Given that this study was conducted post‐COVID‐19, it is plausible that the pandemic contributed to this increase. The Global of Disease Burden study in 2020 documented a 27.6% global increase in MDD cases attributed to COVID‐19 [37].

Assessment of anhedonia, a key diagnostic criterion for patients with MDD [12], is challenging. However, several measures have been developed to assess different aspects of anhedonia in clinical settings. In this study, SHAPS was used as a self‐reported tool to identify MDD patients exhibiting anhedonia symptoms. SHAPS, though a measure of consummatory pleasure, has been shown to be relevant in the identification of anhedonia in most psychiatric conditions including MDD, where consummatory anhedonia was more commonly detected among individuals who are depressed [38]. Subjects with MDD‐ANH had worse SHAPS and PHQ‐9 scores indicating higher severity of MDD (depression) along with anhedonia or higher disease burden in the MDD‐ANH versus MDD non‐ANH group.

In the present study, the greater sexual functioning disability measured through ASEX scores [23] were mediated through anhedonia wherein respondents with MDD‐ANH had significantly worse sexual dysfunction than those with MDD non‐ANH. These findings were in line with the clinical presentation of individuals exhibiting anhedonia symptoms, thus reinforcing the use of SHAPS as a reliable screening instrument for evaluating anhedonia among MDD patients [15, 20].

Furthermore, the results demonstrated that MDD‐ANH respondents (vs. MDD non‐ANH) had higher patient‐centric burden as indicated by significantly lower mental and physical health functioning, worse mobility, self‐care, usual activities, pain/discomfort /EQ‐VAS, and anxiety (GAD‐7) (all *p* < 0.001), indicating the negative impact of anhedonia on HRQoL and anxiety in patients with MDD‐ANH. Additionally, the mediation analysis revealed that a significant proportion of HRQoL parameters (MCS/PCS/EQ‐5D, VAS scale) and anxiety were influenced by anhedonia, highlighting its negative impact on MDD outcomes. Literature spanning across a decade and including all age groups revealed that patients with MDD exhibit poor HRQoL in terms of interpersonal, psychological, and even physical functioning [39, 40, 41]. Also, the presence of anhedonia may increase the severity of MDD as it exerts severe distress [14] and is associated with poor HRQoL in patients with MDD [13]. A large prospective cohort study reported anhedonia as the strongest predictor of poor psychosocial functioning in MDD‐ANH patients [9], leading to higher overall disease severity [12].

In the current study, respondents with MDD‐ANH had significantly higher clinical burden (demonstrated by psychiatrist visits) than MDD non‐ANH respondents (all *p* < 0.001). In addition, a significant proportion (~12%) of psychiatrists' visits (as an MDD outcome) were mediated through anhedonia (*p* < 0.05). Although the studies assessing the direct effect of MDD‐ANH on HRU are limited, the above findings could be attributed to the mounting disease burden of anhedonia to MDD in terms of worse response to treatment and worsening of symptoms with the disease severity [42].

In our study, respondents with MDD‐ANH (vs. MDD non‐ANH) had a greater economic burden as indicated by significantly greater absenteeism, greater overall work productivity impairment, and daily activity impairment, all of which were mediated through anhedonia (all *p* < 0.05). A database study on patients with MDD‐ANH showed high absenteeism, unemployment, and disability rates with the work status being negatively impacted by the presence of anhedonia [43]. Also, MDD contributes to direct and indirect costs associated with health‐care/work productivity [44, 45], especially in the post‐COVID era [7]. High workplace‐related costs are recognized as a major economic burden (owing to loss of productivity), especially in the young and employed population with MDD [6].

Overall, this study used self‐reported patient‐reported outcomes to investigate the impact of anhedonia among individuals with MDD. The study provided insights into the prevalence of MDD and anhedonia in Asia–Pacific which was a similar landscape to the global estimate. The study findings also revealed that the presence of anhedonia in MDD contributed significantly to a greater overall disease burden attributed to MDD in Asia–Pacific, with the potential to incur enormous costs on health care, patients, and caregivers. This indicates a need for future efforts to address the needs of subpopulations and improve population health outcomes.

## 5 Limitations

The cross‐sectional nature of this study does not allow any causal inferences to be drawn. This study allows insight into these different interpretations, but a longitudinal study would be needed to assess bidirectional and temporal effects. The survey is broadly representative of the corresponding national adult population in the respective countries/territories; however, as the questionnaire was administered online, respondents without internet access or those not comfortable with online administration may have been under‐represented. Further, the study also included the general population. We used age‐ and gender‐weighting based on the United Nations population estimates. However, we acknowledge the limitation that, as an online study, it may not be representative of older individuals, those who were institutionalized, or those with lower education levels and may have limited the generalizability of the results. Due to the self‐reported nature of the diagnosis (which cannot be verified), the results may be affected by recall bias. Further, it is important to acknowledge that factors such as geography, socioeconomic status, and MDD severity may influence the generalizability of the findings and would warrant further investigations. As this study focused on investigating the impact of anhedonia among individuals with MDD, the presence of anhedonia was not assessed among the general population which may influence the patient‐reported outcomes within the general population. There are many aspects of anhedonia that may be differentially detected by different scales or instruments. As SHAPS measures the consummatory aspects of anhedonia and not the anticipatory counterpart of anhedonia, the study findings may be limited in considering the multidimensional aspects of anhedonia.

## 6 Conclusions

The findings indicate that MDD patients with anhedonia have a significant disease burden with significantly lower HRQoL, higher WPAI, and more HRU than MDD patients without anhedonia, emphasizing the need for better diagnosis and treatment interventions to mitigate the burden of anhedonia on MDD patients in the Asia–Pacific region.

## Author Contributions

K.H.: conceptualization, methodology, validation, resources, writing – review and editing, supervision, project administration, funding acquisition; M.B.: methodology, validation, resources, writing – review and editing; W.‐L.H.: methodology, validation, resources, writing – review and editing; T.K.: methodology, validation, resources, writing – review and editing; J.G.L.: methodology, validation, resources, writing – review and editing; C.G.N.: methodology, validation, resources, writing – review and editing; Z.W.: methodology, validation, resources, writing – review and editing; T.W.: methodology, validation, resources, writing – review and editing; M.K.‐K.: validation, resources, writing – review and editing, funding acquisition; L.V.: conceptualization, methodology, validation, formal analysis, investigation, resources, data curation, writing – review and editing, visualization, supervision, project administration.

## Ethics Statement

The study was conducted as per the Declaration of Helsinki and in line with Good Epidemiological Practices as outlined by the International Society for Pharmacoeconomics and Outcomes Research. The study protocol and study material (questionnaire) were submitted to and approved by the Toukekai Kitamachi Clinic Ethics Review Board (ERB) (approval number: EJP09413) for the conduct of the study in Japan. The study protocol (along with study material [questionnaire]) was submitted to the Pearl Institutional Review Board (for conduct of the study in Australia, China Mainland, Malaysia, South Korea, and Taiwan) and was determined to be exempt as per regulations: 45 CRF 46.104(b) (2) Tests, Surveys, Interviews (IRB number: 023‐0025). Only deidentified data was collected and analyzed.

## Consent

Informed consent was obtained from all participants of the study.

## Conflicts of Interest

M.B. is supported by a NHMRC Senior Principal Research Fellowship and Leadership 3 Investigator grant (1156072 and 2017131). W.‐L.H. has received consultation fees from Janssen, Servier, and Boehringer Ingelheim. He has given lectures with personal honoraria for Janssen, Servier, Pfizer/Viatris, Sumitomo, Otsuka, and Boehringer Ingelheim. T.K. reports personal fees from Janssen Asia Pacific/Vista Health related to this work, reports grants from Sumitomo Pharma Co. Ltd., Otsuka Pharmaceutical Co. Ltd., Takeda Pharmaceutical Co. Ltd., Eli Lilly Japan K.K., Teijin Pharma, Daiichi Sankyo Co. Ltd., EA Pharma Co. Ltd., and Eisai Co. Ltd., personal fees from Sumitomo Pharma Co. Ltd., Otsuka Pharmaceutical Co. Ltd., Takeda Pharmaceutical Co. Ltd., Eisai Co. Ltd., Meiji Seika Pharma Co. Ltd., Shionogi & Co. Ltd., Mochida Pharmaceutical Co., Janssen Pharmaceutical K.K., Janssen Asia Pacific, Vista Health, Yoshitomiyakuhin, MSD K.K., Japan Boehringer Ingelheim, Kyowa Pharmaceutical Industry Co. Ltd., Viatris, Mylan EPD, H.U. Frontier, Lundbeck Japan K.K., Nihon Medi‐physics Co. Ltd., Glaxo‐SmithKline, Novartis Pharma, EA Pharma Co., and Ono Pharmaceutical Co. Ltd., outside the submitted work. L.V. is an employee of Oracle Life Sciences, Singapore. K.H. and T.W. are employees of Janssen Asia Pacific, a division of Johnson & Johnson Pte Ltd., Singapore. M.K.‐K. is an employee of Janssen Pharmaceutical K.K., Tokyo, Japan. J.G.L., C.G.N., and Z.W. declare no conflicts of interest.

## Supporting information


Data S1.

## Data Availability

Participants in this study had only provided informed consent to sharing analyzed aggregated data, not individual‐level data (raw data), even if anonymized. The raw data requires cross‐referencing with the survey questionnaire which cannot be shared publicly due to licensing restrictions as the survey questionnaire includes licensed patient‐reported outcome measures. The data that support the findings of this study are available from the corresponding author upon reasonable request.
