# Supplementary material for: The Impact of Anhedonia on the Disease Burden of Major Depressive Disorder in the Asia–Pacific Region: A Cross‐Sectional Real‐World Study
Source: Neuropsychopharmacol Rep. 2025 Feb 26;45(1):e70007. doi: 10.1002/npr2.70007 (PMC11864854; doi:10.1002/npr2.70007)
Supplement: Supplementary file 1 — Data S1. [file NPR2-45-e70007-s001.docx]

**Supplementary material**

**Supplementary Table 1. Multivariate analysis.**

| **Multivariate analysis** | A modified version of PHQ-9 was included in the regressions to adjust for depression severity. The anhedonia-specific item of PHQ-9 (item 1) was removed to avoid collinearity. The remaining items of PHQ-9 were summed as usual in PHQ-9 and included in the regression as such. The regression results with the anhedonia as a categorical variable focused on comparing MDD-ANH and MDD non-ANH. General Population was excluded from the comparison due to differing PHQ-9 inclusion criteria between General Population and the MDD patients. |
| --- | --- |
| **Mediation analysis** | The **mediator model** measured whether PHQ-9 and SHAPS correlate which is a prerequisite for SHAPS mediating an effect through PHQ-9. The **outcome model** indicated whether SHAPS and PHQ-9 have an effect on the outcome. The **mediation analysis** quantified the total effect that PHQ-9 and SHAPS (combined) have on MDD outcomes. The **average mediation effect** measured the amount of the total SHAPS and PHQ-9 effect is mediated through SHAPS. The **proportion mediated** was the proportion of the total effect of SHAPS and PHQ-9 that was mediated through SHAPS |
| **Missing data details** | Some questions may not be answered by all respondents because they are only applicable to certain respondents (e.g., questions about current prescription treatment for depression are only shown to respondents who reported currently using a prescription for depression). For these questions where respondents are not included in the denominator, the valid N will be reported. Decline to answer options are provided for items asking about potentially sensitive topics, such as weight or income. For such questions, the frequency and percentage of those who declined to answer will be reported. |

Abbreviations: ANH, anhedonia; MDD, major depressive disorder; MDD-ANH, MDD with anhedonia; MDD non-ANH, MDD without anhedonia; PHQ-9, Patient Health Questionnaire-9-item; SHAPS, Snaith-Hamilton Pleasure Scale

**Supplementary Table 2. Sociodemographic and health characteristics of the MDD-ANH, MDD non-ANH, and General Population.**

|  | **MDD-ANH**  **(n = 1448)** | **MDD non-ANH**  **(n = 836)** | **General Population**  **(n = 9099)** |
| --- | --- | --- | --- |
|  | n (%) | n (%) | n (%) |
| ***S1. Country*** |  |  |  |
| Australia | 241 (16.6) | 156 (18.7) | 1402 (15.4) |
| China | 234 (16.2) | 163 (19.5) | 1562 (17.2) |
| Japan | 307 (21.2) | 54 (6.5) | 1575 (17.3) |
| Malaysia | 214 (14.8) | 186 (22.3) | 1406 (15.5) |
| South Korea | 260 (18.0) | 119 (14.2) | 1571 (17.3) |
| Taiwan | 192 (13.3) | 158 (18.9) | 1583 (17.4) |
| ***S2. Race*** |  |  |  |
| Black | 11 (0.8) | 6 (0.7) | 18 (0.2) |
| White | 205 (14.2) | 140 (16.8) | 1251 (13.8) |
| Hispanic/ Latino | 7 (0.5) | 1 (0.1) | 13 (0.1) |
| Middle Eastern | 9 (0.6) | 2 (0.2) | 21 (0.2) |
| Chinese | 462 (31.9) | 339 (40.6) | 3560 (39.1) |
| Japanese | 306 (21.1) | 56 (6.7) | 1581 (17.4) |
| Korean | 259 (17.9) | 117 (14.0) | 1573 (17.3) |
| Indian | 22 (1.5) | 12 (1.4) | 90 (1.0) |
| Filipino | 1 (0.1) | 6 (0.7) | 32 (0.4) |
| Malay | 159 (11.0) | 141 (16.9) | 826 (9.1) |
| Other Asian | 23 (1.6) | 16 (1.9) | 149 (1.6) |
| Others | 19 (1.3) | 15 (1.8) | 80 (0.9) |
| ***S3. BMI Categories*** |  |  |  |
| Underweight (< 18.5) | 199 (13.7) | 114 (13.6) | 774 (8.5) |
| Normal weight (18.5 to < 25) | 784 (54.1) | 488 (58.4) | 5626 (61.8) |
| Overweight (25 to < 30) | 295 (20.4) | 121 (14.5) | 1890 (20.8) |
| Obese (30 or greater) | 170 (11.7) | 113 (13.5) | 809 (8.9) |
| Unknown | 0 (0.0) | 0 (0.0) | 0 (0.0) |
| ***S4. Frequency of smoking*** |  |  |  |
| Every day | 495 (34.2) | 273 (32.7) | 1980 (21.8) |
| Some days | 319 (22.0) | 228 (27.3) | 1218 (13.4) |
| Not at all | 634 (43.8) | 335 (40.1) | 5901 (64.9) |
| ***S5. Frequency of consuming alcohol*** |  |  |  |
| Every day | 270 (18.7) | 119 (14.2) | 1118 (12.3) |
| Some days | 786 (54.3) | 506 (60.5) | 5088 (55.9) |
| Not at all | 392 (27.1) | 211 (25.2) | 2893 (31.8) |
| ***S6. Frequency of doing exercise a week*** |  |  |  |
| More than 5 times a week | 125 (8.6) | 145 (17.3) | 1828 (20.1) |
| 3 to 5 times a week | 324 (22.4) | 262 (31.3) | 2563 (28.2) |
| 1 to 2 times a week | 469 (32.4) | 288 (34.5) | 2656 (29.2) |
| Very rarely or never | 530 (36.6) | 141 (16.9) | 2052 (22.6) |

Abbreviations: ANH, anhedonia; MDD, major depressive disorder; BMI, body mass index; MDD-ANH, MDD with anhedonia; MDD non-ANH, MDD without anhedonia.

**Supplementary Table 3. Multivariable results for patient-centric, economic, and clinical outcomes (MDD-ANH versus MDD non-ANH versus General Population).**

| **Outcomes** | **MDD-Anhedonia Groups** | | |  |
| --- | --- | --- | --- | --- |
| ***Sexual functioning*** | **Group^a^** | **Adjusted mean** | **Odds ratio (95% CI)** | ***P* value^b^** |
| Arizona Sexual Health Scale^c^ | MDD-ANH | 0.57 | 5.22 (4.53 – 6.03) | < 0.001 |
|  | MDD non-ANH | 0.35 | 2.11 (1.76 – 2.53) | < 0.001 |
|  | General Population | 0.20 |  |  |
| ***HRQoL*** | **Group^a^** | **Adjusted mean** | **Beta-coefficient (95% CI)** | ***P* value^b^** |
| RAND - MCS^d^ | MDD-ANH | 27.48 | -22.49 (-22.96 – -22.02) | < 0.001 |
|  | MDD non-ANH | 31.50 | -18.46 (-19.06 – - 17.87) | < 0.001 |
|  | General Population | 49.97 |  |  |
| RAND – PCS^d^ | MDD-ANH | 35.56 | -15.00 (-15.42 – - 14.58) | < 0.001 |
|  | MDD non-ANH | 37.00 | -13.56 (-14.10 – - 13.03) | < 0.001 |
|  | General Population | 50.56 |  |  |
| EQ-5D Index score^d^ | MDD-ANH | 0.68 | -0.24 (-0.24 – - 0.23) | < 0.001 |
|  | MDD non-ANH | 0.74 | -0.18 (-0.19 – -0.18) | < 0.001 |
|  | General Population | 0.92 |  |  |
| EQ-VAS score^d^ | MDD-ANH | 52.00 | -29.20 (-30.10 – - 28.29) | < 0.001 |
|  | MDD non-ANH | 62.93 | -18.26 (-19.40 – - 17.13) | < 0.001 |
|  | General Population | 81.20 |  |  |
| ***Mental Health^d^*** | **Group^a^** | **Adjusted mean** | **Beta-coefficient (95% CI)** | ***P* value^b^** |
| PHQ-9 score | MDD-ANH | 16.373 | 14.577 | 14.439 |
|  | MDD non-ANH | 14.743 | 12.948 | 12.774 |
|  | General Population | 1.795 |  |  |
| GAD-7 score | MDD-ANH | 12.44 | 10.77 (10.61 – 10.92) | < 0.001 |
|  | MDD non-ANH | 10.88 | 9.21 (9.01 – 9.41) | < 0.001 |
|  | General Population | 1.68 |  |  |
| ***Labor Force Participation^c^*** | **Group** | **Adjusted mean** | **Odds ratio (95% CI)** | ***P* value^b^** |
| Participating in the labor force^g^ | MDD-ANH (n=1404) | 0.86 | 0.50 (0.41 – 0.61) | < 0.001 |
|  | MDD non-ANH (n=815) | 0.90 | 0.75 (0.56 – 1.01) | 0.055 |
|  | General Population (n=7863) | 0.93 |  |  |
| ***WPAI*** | **Group** | **Adjusted mean** | **Rate ratio (95% CI)** | ***P* value^b^** |
| Absenteeism^e^ | MDD-ANH (n=1107) | 14.35 | 3.12 (2.84 – 3.43) | < 0.001 |
|  | MDD non-ANH (n=683) | 11.77 | 2.56 (2.30 – 2.84) | < 0.001 |
|  | General Population (n=6882) | 4.60 |  |  |
| Presenteeism^d^ | MDD-ANH (n=1090) | 56.94 | 36.30 (34.52 – 38.08) | < 0.001 |
|  | MDD non-ANH (n=679) | 52.87 | 32.23 (30.04 – 34.41) | < 0.001 |
|  | General Population (n=6866) | 20.64 |  |  |
| Overall Work Productivity Impairment^d^ | MDD-ANH (n=1090) | 62.09 | 39.06 (37.22 – 40.91) | < 0.001 |
|  | MDD non-ANH (n=679) | 57.24 | 34.22 (31.95 – 36.49) | < 0.001 |
|  | General Population (n=6866) | 23.02 |  |  |
| Activity Impairment^d^ | MDD-ANH (n=1448) | 57.15 | 37.90 (36.41 – 39.39) | < 0.001 |
|  | MDD non-ANH (n=836) | 51.70 | 32.45 (30.58 – 34.33) | < 0.001 |
|  | General Population (n=9099) | 19.25 |  |  |
| ***Healthcare Resource Use^f^*** | **Group^a^** | **Adjusted mean** | **Rate ratio (95% CI)** | ***P* value^b^** |
| # of GP visits in the past 6 months | MDD-ANH | 0.44 | 2.51 (2.05 – 3.08) | < 0.001 |
|  | MDD non-ANH | 0.41 | 2.35 (1.81 – 3.05) | < 0.001 |
|  | General Population | 0.18 |  |  |
| # of ER visits in past 6 months | MDD-ANH | 0.66 | 3.52 (2.71 – 4.58) | < 0.001 |
|  | MDD non-ANH | 0.55 | 2.96 (2.14 – 4.10) | < 0.001 |
|  | General Population | 0.19 |  |  |
| # of Hospitalizations in past 6 months | MDD-ANH | 0.22 | 2.74 (2.12 – 3.54) | < 0.001 |
|  | MDD non-ANH | 0.19 | 2.34 (1.72 – 3.18) | < 0.001 |
|  | General Population | 0.08 |  |  |
| # of Psychiatrist visits in past 6 months | MDD-ANH | 0.82 | 90.71 (55.27 – 148.90) | < 0.001 |
|  | MDD non-ANH | 0.46 | 50.40 (28.58 – 88.89) | < 0.001 |
|  | General Population | 0.01 |  |  |
| # of Psychologist/therapist visits in past 6 months | MDD-ANH | 0.37 | 35.74 (23.51 – 54.33) | < 0.001 |
|  | MDD non-ANH | 0.28 | 27.39 (16.58 – 45.24) | < 0.001 |
|  | General Population | 0.01 |  |  |

^a^MDD-ANH (n=1448); MDD non-ANH (n=836); General Population (n=9099)

^b^Note: *P* value was calculated based on the comparison with General Population. The analysis was performed controlling for country, race, sex, age, BMI, frequency of smoking, frequency of consuming alcohol, frequency of exercise, education, employment status, CCI

^c^Binary logistic regression model

^d^GLM w/ normal distribution and identity link

^e^GLM w/ normal distribution and log link for Absenteeism and Presenteeism, Overall Work Productivity Impairment, Activity Impairment with identify link function

^f^GLM w/ negative binomial distribution and log-link

^g^Covariates restricted to age, sex, race, and CCI due to convergence issues

^#^Number

Abbreviations: ANH, anhedonia; CCI, Charlson comorbidity index; CI, confidence interval; EQ-5D-5L, EuroQol 5 Dimension Health Questionnaire; ER, emergency room; GAD-7, 7-item Generalized Anxiety Disorder Assessment scale; GP, general physician; MCS, mental component summary; MDD, major depressive disorder; MDD-ANH, MDD with anhedonia; MDD non-ANH, MDD without anhedonia; PCS, physical component summary; VAS, visual analogue scale; WPAI, Work productivity and activity impairment.

**Supplementary Table 4. Mediation analysis for patient-centric outcomes: Mental health, HRQoL, and sexual functioning ASEX scores.**

| **Mediator Model**  **(N = 2284)** | **GAD-7^a^** | | **RAND MCS^a^** | | **RAND PCS^a^** | | **EQ-5D Index^a^** | | **EQ-VAS^a^** | | **ASEX Scale^b^** | |
| --- | --- | --- | --- | --- | --- | --- | --- | --- | --- | --- | --- | --- |
|  | Coefficient | *P* value | Coefficient | *P* value | Coefficient | *P* value | Coefficient | *P* value | Coefficient | *P* value | Coefficient | *P* value |
| PHQ-9 | 0.255 | < 0.001 | 0.255 | < 0.001 | 0.255 | < 0.001 | 0.255 | < 0.001 | 0.255 | < 0.001 | 0.255 | < 0.001 |
| **Outcome Model** | | | | | | | | | | | | |
| SHAPS | 0.099 | < 0.001 | -0.479 | < 0.001 | -0.216 | < 0.001 | -0.006 | < 0.001 | -1.468 | < 0.001 | 0.146 | < 0.001 |
| PHQ-9 | 0.671 | < 0.001 | -0.662 | < 0.001 | -0.503 | < 0.001 | -0.012 | < 0.001 | -1.004 | < 0.001 | 0.052 | < 0.001 |
| **Mediation analysis** | | | | | | | | | | | | |
| Total Effect | 0.696 | < 0.001 | -0.785 | < 0.001 | -0.558 | < 0.001 | -0.014 | < 0.001 | -1.381 | < 0.001 | 0.153 | < 0.001 |
| Average Mediation Effect | 0.025 | < 0.001 | -0.112 | < 0.001 | -0.055 | < 0.001 | -0.002 | < 0.001 | -0.378 | < 0.001 | 0.064 | < 0.001 |
| Average Direct Effect | 0.671 | < 0.001 | -0.662 | < 0.001 | -0.503 | < 0.001 | -0.012 | < 0.001 | -1.004 | < 0.001 | 0.089 | < 0.001 |
| **Proportion Mediated** | **0.036** | **< 0.001** | **0.156** | **< 0.001** | **0.099** | **< 0.001** | **0.117** | **< 0.001** | **0.273** | **< 0.001** | **0.417** | **< 0.001** |

Note: controlling for country, age, sex, and CCI

^a^GLM w/ normal distribution and identity link

^b^Binary logistic regression model

Abbreviations: ASEX, Arizona Sexual Experience Scale; CCI, Charlson comorbidity index; EQ-5D-5L, EuroQol 5 Dimension Health Questionnaire; GAD-7, 7-item Generalized Anxiety Disorder Assessment scale; GLM, generalized linear model; HRQoL, health-related quality of life; MCS, mental component summary; PCS, physical component summary; PHQ-9, Patient Health Questionnaire-9-item; SHAPS, Snaith-Hamilton Pleasure Scale; VAS, visual analogue scale.

**Supplementary Table 5. Mediation analysis for economic outcomes: Labor force participation and WPAI.**

| **Mediator Model** | **Labor Force Participation^a^**  **(N = 2219)** | | **Absenteeism^b^**  **(N = 1790)** | | **Presenteeism^c^**  **(N = 1769)** | | **WPAI^c^**  **(N = 1769)** | | **Activity Impairment^c^ (N = 2284)** | |
| --- | --- | --- | --- | --- | --- | --- | --- | --- | --- | --- |
|  | Coefficient | *P* value | Coefficient | *P* value | Coefficient | *P* value | Coefficient | *P* value | Coefficient | *P* value |
| PHQ-9 | 0.251 | < 0.001 | 0.254 | < 0.001 | 0.251 | < 0.001 | 0.251 | < 0.001 | 0.255 | < 0.001 |
| **Outcome Model** | Coefficient | *P* value | Coefficient | *P* value | Coefficient | *P* value | Coefficient | *P* value | Coefficient | *P* value |
| SHAPS | -0.054 | 0.002 | 0.015 | 0.030 | 0.527 | < 0.001 | 0.609 | < 0.001 | 0.608 | < 0.001 |
| PHQ-9 | -0.051 | 0.001 | 0.020 | 0.003 | 1.141 | < 0.001 | 1.079 | < 0.001 | 1.184 | < 0.001 |
| **Mediation analysis** | Coefficient | *P* value | Coefficient | *P* value | Coefficient | *P* value | Coefficient | *P* value | Coefficient | *P* value |
| Total Effect | -0.058 | < 0.001 | 0.325 | < 0.001 | 1.273 | < 0.001 | 1.232 | < 0.001 | 1.339 | < 0.001 |
| Average Mediation Effect | -0.012 | < 0.001 | 0.052 | < 0.001 | 0.132 | < 0.001 | 0.153 | < 0.001 | 0.155 | < 0.001 |
| Average Direct Effect | -0.046 | < 0.001 | 0.273 | 0.020 | 1.141 | < 0.001 | 1.079 | < 0.001 | 1.184 | < 0.001 |
| **Proportion Mediated** | **0.210** | **< 0.001** | **0.162** | **< 0.001** | **0.104** | **< 0.001** | **0.124** | **< 0.001** | **0.116** | **< 0.001** |

Note: controlling for country, age, sex, and CCI; Item 1 (Anhedonia) was removed from PHQ-9 to avoid collinearity between outcome and primary independent variable

^a^Binary logistic regression model

^b^GLM w/ normal distribution and log link

^c^GLM w/ normal distribution and identity link

Abbreviations: CCI, Charlson comorbidity index; GLM, generalized linear model; PHQ-9, Patient Health Questionnaire-9-item; SHAPS, Snaith-Hamilton Pleasure Scale; WPAI, Work productivity and activity impairment.

**Supplementary Table 6. Mediation analysis for clinical outcomes: Health resource utilization.**

| **Mediator Model**  **(N = 2284)** | **# of GP visits^a^** | | **# of ER visits^a^** | | **# of Hospitalizations^a^** | | **# of Psychiatrist visits^a^** | | **# of Psychologist visits^a^** | |
| --- | --- | --- | --- | --- | --- | --- | --- | --- | --- | --- |
|  | Coefficient | *P value* | Coefficient | *P* value | Coefficient | *P value* | Coefficient | *P value* | Coefficient | *P value* |
| PHQ-9 | 0.255 | < 0.001 | 0.255 | < 0.001 | 0.255 | < 0.001 | 0.255 | < 0.001 | 0.255 | < 0.001 |
| **Outcome Model** | Coefficient | *P* value | Coefficient | *P* value | Coefficient | *P* value | Coefficient | *P* value | Coefficient | *P* value |
| SHAPS | -0.010 | 0.544 | 0.011 | 0.358 | -0.0001 | 0.994 | 0.041 | 0.007 | 0.014 | 0.459 |
| PHQ-9 | 0.030 | 0.041 | 0.017 | 0.147 | 0.031 | 0.019 | 0.078 | < 0.001 | 0.054 | 0.004 |
| **Mediation analysis** | Coefficient | *P* value | Coefficient | *P* value | Coefficient | *P* value | Coefficient | *P* value | Coefficient | *P* value |
| Total Effect | 0.017 | 0.100 | 0.017 | 0.120 | 0.010 | < 0.001 | 0.029 | < 0.001 | 0.016 | < 0.001 |
| Average Mediation Effect | -0.002 | 0.600 | 0.002 | 0.400 | -0.00001 | 0.920 | 0.003 | 0.040 | 0.001 | 0.440 |
| Average Direct Effect | 0.018 | 0.060 | 0.014 | 0.260 | 0.010 | 0.040 | 0.026 | < 0.001 | 0.015 | < 0.001 |
| **Proportion Mediated** | **-0.091** | **0.700** | **0.142** | **0.480** | **-0.001** | **0.920** | **0.119** | **0.040** | **0.064** | **0.440** |

Note: controlling for country, age, sex, and CCI

^a^GLM w/ negative binomial distribution and log-link

Abbreviations: CCI, Charlson comorbidity index; ER, emergency room; GLM, generalized linear model; GP, general physician; PHQ-9, Patient Health Questionnaire-9-item; SHAPS, Snaith-Hamilton Pleasure Scale.

**Supplementary Figure 1. Screening of participants invited to the study according to the inclusion and exclusion criteria**

**
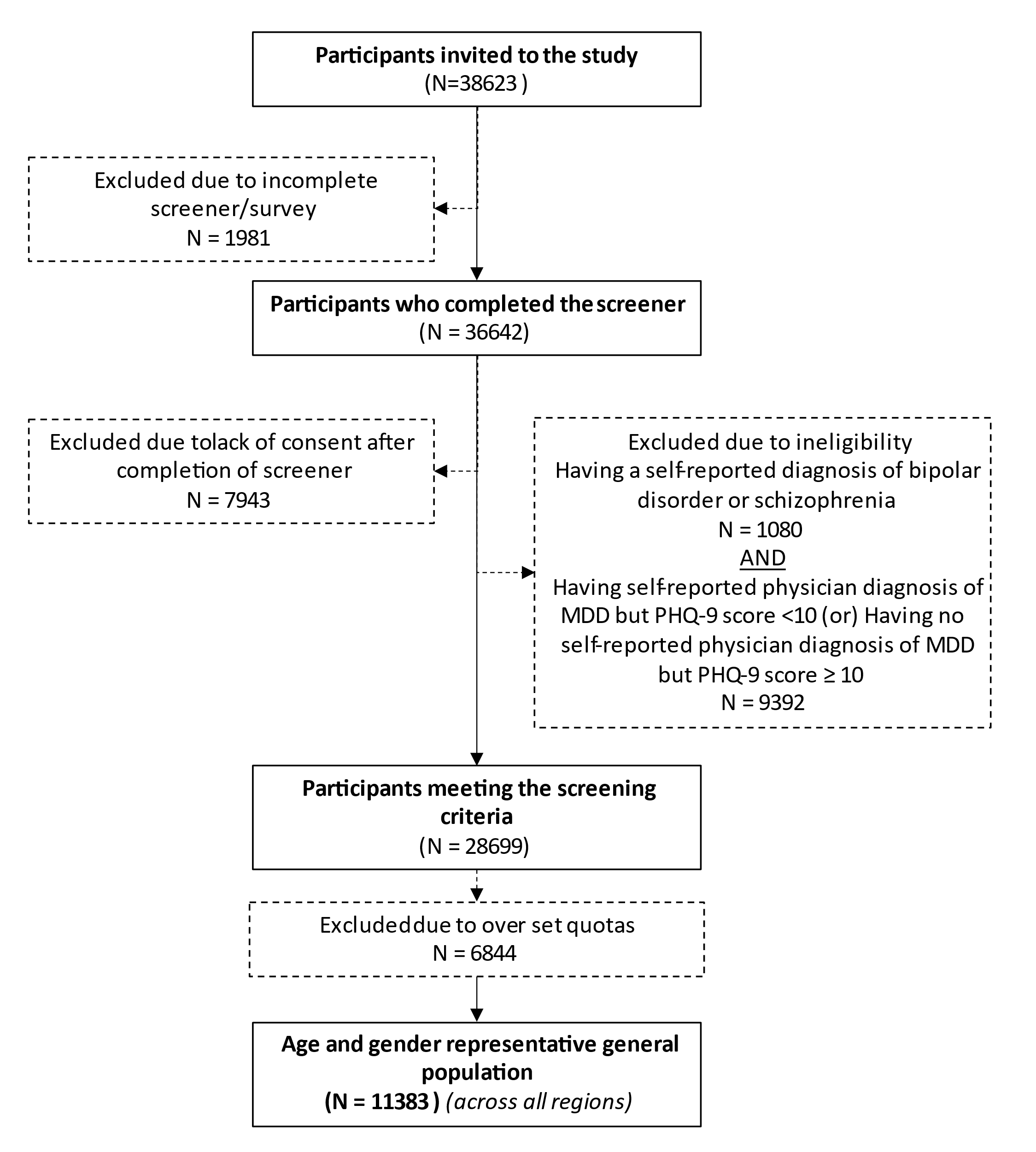
**

Abbreviations: MDD, major depressive disorder; PHQ-9, Patient Health Questionnaire-9-item

**Supplementary Figure 2. Mediation analysis variables.**

**
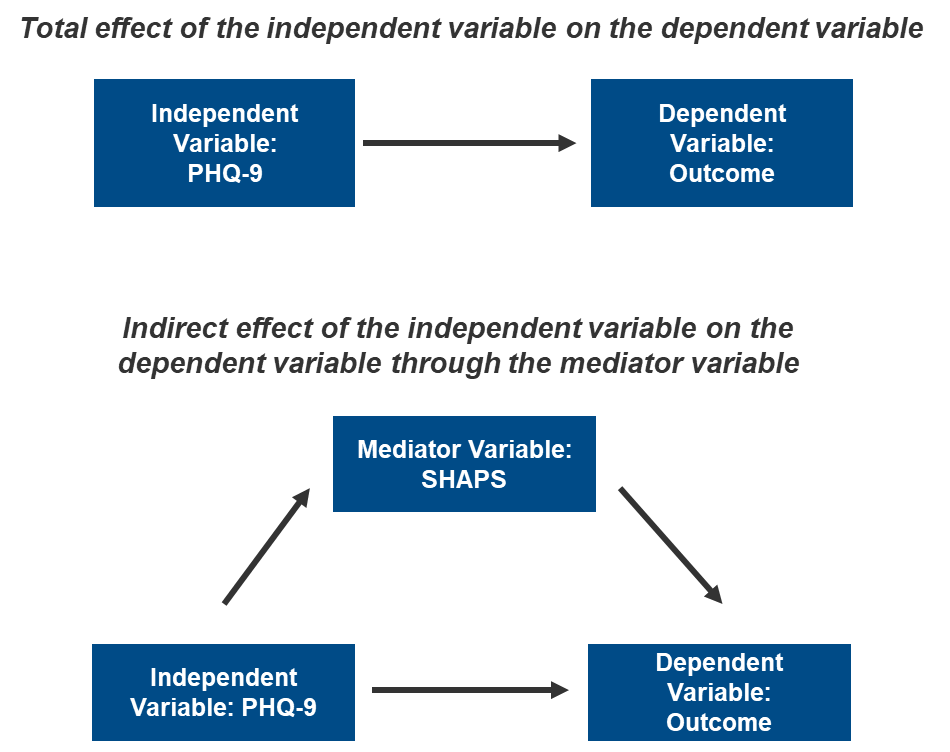
**

Abbreviations: Patient Health Questionnaire-9-item; SHAPS, Snaith-Hamilton Pleasure Scale.
